# Targeting HypoxamiRs: A new perspective on overcoming treatment resistant glioblastoma

**DOI:** 10.1016/j.omtn.2025.102753

**Published:** 2025-11-03

**Authors:** Rajat Choudhary, Debyashreeta Barik, V Badireenath Konkimalla

**Affiliations:** 1 School of Biological Sciences, National Institute of Science Education and Research, HBNI, Jatni, Odisha 752050, India; 2 Homi Bhabha National Institute, Training School Complex, Anushakti Nagar, Mumbai 400094, India

## Main text

Glioblastoma and high-grade gliomas are among the most aggressive central nervous system tumors, where hypoxia-driven pathways dictate progression and resistance. Hypoxia sustains glioma stem-like cells, promotes angiogenesis, and creates an immunosuppressive microenvironment. At the core is hypoxia-inducible factor-1 α (HIF-1α), a transcriptional regulator that activates survival and invasion processes. A key mechanism involves hypoxia-responsive microRNAs, or hypoxamiRs, which fine-tune gene expression to support tumor adaptation. Both induced and suppressed hypoxamiRs reshape the transcriptional landscape, linking to glioblastoma pathobiology.^1^^,^^2^

**Figure 1:**
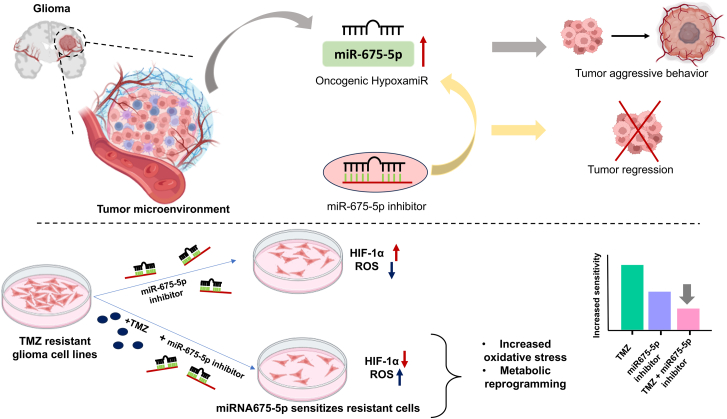
A schematic illustration of miR-675-5p′s function as hypoxamiR in glioma and temozolomide (TMZ) resistance; miRNA-675-5p, an oncogenic hypoxamiR, promotes HIF-1α stability, ROS production, and tumor aggressiveness in hypoxic glioma microenvironment Inhibition of miR-675-5p reduces HIF-1α and ROS levels, which promotes tumor regression (above). In TMZ-resistant glioma cell lines, using a miR-675-5p inhibitor with TMZ improves drug susceptibility by increasing oxidative stress and triggering metabolic reprogramming (below).

Targeting the HIF-1α-hypoxamiR axis could potentially disrupt tumor-promoting pathways, reduce stemness, and enhance therapy response.^3^ Among miRNAs, miRNA675-5p (hypoxamiR) represents a promising candidate, linking hypoxic signaling to tumor survival and treatment evasion against 5-fluorouracil-resistant colorectal cancer.^4^^,^^5^ In a recent study published in Molecular Therapy Nucleic Acids, Martelli C. et al.^6^ and colleagues demonstrated that inhibition of hypoxia-induced miRNA-675-5p activity in temozolomide (TMZ)-resistant glioblastoma, subverted therapy resistance and targeted residual cells driving relapse. Integrating miRNA675-5p-based intervention, complemented with current TMZ (a standard drug of GBM therapy), can improve patient outcome; however, such studies are not thoroughly explored. This way, inhibiting HIF-1α activity or modulating hypoxamiRs could disrupt tumor-promoting pathways, reduce maintenance of glioma stem cells, and enhance the efficacy of existing TMZ-resistant glioma therapies, proving a rational framework for future treatment approaches. (Figure 1).

This study demonstrated that miRNA675-5p inhibition induces a robust antitumor response in glioma cells irrespective of their TMZ sensitivity wherein the cell viability is reduced, energy production is impaired activating apoptotic pathways. The upregulation of pro-apoptotic genes, suppression of survival signals, and an active caspase-mediated death highlight a coordinated collapse of tumor supportive mechanisms. Central to this effect is the downregulation of HIF-1α, a key transcription factor driving hypoxia-mediated tumor progression. Stripped of its nuclear presence, this transcription factor loses its power to sustain the malignant program. For resistant glioma, long accustomed to thriving despite chemotherapy, this disruption represents a critical vulnerability. This disruption extends MAPK and TGF-β signaling becoming subdued, cutting off fuel for the epithelial-to-mesenchymal transition that empowers glioma cells to invade the surrounding brain. Furthermore, the cellular skeleton itself shifts, altering the architecture and slowing migration. Together, these findings position miRNA675-5p as a key regulator of glioma aggressiveness; upon its inhibition not only comprises tumor survival but also reverses malignant features, suggesting a promising therapeutic avenue, particularly overcoming resistance to conventional chemotherapy.

A significant effect of miRNA675-5p inhibition is the induction of oxidative stress, characterized by a surge in reactive oxygen species (ROS) that overwhelms the compromised antioxidant defense system. Expression of key redox enzymes, including superoxide dismutase and catalase, is reduced, and NRF2 activity is suppressed due to increased KEAP1 expression. Inhibition of detoxification pathways elevates oxidative stress beyond the cells’ survival threshold. The reversal of inhibitor-induced cell death by mitochondrial ROS scavenging confirms the causal role of redox imbalance. This treatment strategy effectively induces glioma cell destruction by disrupting redox homeostasis.

The biochemical fingerprints of this imbalance were well-noted in glutathione metabolism where under such conditions, the cells try to make more glutathione, but the oxidized form builds up at the same time, indicating that the redox system is under stress and not working properly. Sensitive cells exhibit a tendency toward imbalance, whereas resistant cells demonstrate a more intricate, stable response, potentially indicative of their extensive experience in enduring oxidative stress. But the message is clear: the inhibitor makes glioma face a redox landscape that it can’t handle.

Beyond immediate effects, miRNA675-5p inhibition disrupts the metabolic plasticity characteristic of glioma. Silencing this microRNA impairs the pentose phosphate pathway, a primary source of NADPH and nucleotide precursors, and leads to a marked reduction in amino acids essential for redox balance, including serine, glycine, cysteine, and proline. The loss of these metabolic defenses increases cellular vulnerability to oxidative stress by dismantling protective mechanisms.

Biochemical disruption resulting from miRNA675-5p inhibition impedes cell cycle progression, causing accumulation of cells at various checkpoints. This arrest reduces the likelihood of rapid, uncontrolled proliferation. It remains unclear whether this cell cycle stalling represents transient dormancy, which may lead to relapse, or irreversible senescence that limits tumorigenic potential. Regardless, the intervention effectively slows glioblastoma growth.

Nevertheless, glioma’s resilience persists. A new way of using energy starts to form in the cells that survive. When glucose-driven pathways fail, they use glutamine instead. High amounts of glutamine and its byproduct glutamate fuel the tricarboxylic acid cycle and make lactate through glutaminolysis. This change, which is most evident in the resistant cells, demonstrates the flexibility of their metabolism.

The most hopeful events arise when resistant glioma cells, once unresponsive to TMZ, are given another chance. Following miRNA675-5p inhibition and a brief recovery, re-exposure to the drug proves fatal, where viability drops sharply, apoptotic genes reactivate, and survival pathways collapse. Their resistant identity fades, and they resemble sensitive cells once more. Mechanistically, this reversal centres on HIF-1α: its high expression in resistant cells declines to sensitive levels with the inhibitor, and the combined treatment suppresses it even further, restoring drug sensitivity.

Alongside this, the nutrient-sensing regulator RPTOR declines, especially in resistant cells. Since RPTOR governs the mTORC1 complex, a central node of growth and metabolism, its suppression hints at broader vulnerabilities being unmasked. The intersection of hypoxia signaling, redox imbalance, and nutrient sensing suggests that miRNA675-5p inhibition works not as a blunt tool but as a scalpel, cutting into several survival circuits at once.

Taken together, the findings tell a story of duality. On one hand, miRNA675-5p inhibition exerts direct anti-tumor effects: it diminishes growth, provokes oxidative collapse, dismantles invasiveness, and stalls the cell cycle. On the other hand, it serves as a sensitizer, reopening the therapeutic window for TMZ in tumors that had closed it. It is both a therapy in itself and an enabler of existing chemotherapy.

Furthermore, metabolic rewiring reveals the adaptability of glioma, deciphering the survivability of cells that become more and more reliant on glutamine. This shift could lead to relapse if the problem is left unaddressed. These results point to logical pairings of glutaminase or other metabolic blockers with miRNA675-5p inhibition to prevent escape pathways. Likewise, achieving the right balance between inducing oxidative stress and protecting normal tissue will demand precise *in vivo* optimization.

The conclusion, then, is not merely that miRNA675-5p inhibition is effective, but it reshapes the therapeutic perspective on resistant glioma. With only meagre therapeutic options available for glioma therapy, it would be an advantage if gliomas acquiring resistance could alternate or combine treatments that disrupt the tumor’s hypoxic and redox frameworks, pushing it back into vulnerability. By leveraging the metabolic and molecular shifts induced by this inhibitor, therapy could evolve from conventional chemotherapy toward a more precise dismantling of the tumor’s survival networks.

This study offers both mechanistic insight and therapeutic promise. The dual function of miRNA675-5p inhibition as both a weapon and a key reveals that resistance in glioblastoma need not be permanent. With effective translation, it could form the basis of strategies that exploit glioma’s own adaptability to drive its defeat.

## Acknowledgments

The authors thank NISER for their support. R.C., and D.B., acknowledges NISER, DAE, GoI for providing the research fellowship.

Funding: no particular funding from public, private, or non-profit organizations was obtained.

## Author contributions

R.C., D.B., and V.B.K conceptualized the figure, provided new perspectives, and wrote the commentary.

## Declaration of interests

The authors declare that they have no conflict of interest.
